# Biochemical and Physical Characterisation of Urinary Nanovesicles following CHAPS Treatment

**DOI:** 10.1371/journal.pone.0037279

**Published:** 2012-07-12

**Authors:** Luca Musante, Mayank Saraswat, Elodie Duriez, Barry Byrne, Alessandra Ravidà, Bruno Domon, Harry Holthofer

**Affiliations:** 1 Centre for BioAnalytical Sciences (CBAS), Dublin City University, Dublin, Ireland; 2 Luxembourg Clinical Proteomics Center (LCP), CRP-Santé, Strassen, Luxembourg; Ottawa Hospital Research Institute, Canada

## Abstract

Urinary exosomes represent a precious source of potential biomarkers for disease biology. Currently, the methods for vesicle isolation are severely restricted by the tendency of vesicle entrapment, *e.g.* by the abundant Tamm-Horsfall protein (THP) polymers. Treatment by reducing agents such as dithiothreitol (DTT) releases entrapped vesicles, thus increasing the final yield. However, this harsh treatment can cause remodelling of all those proteins which feature extra-vesicular domains stabilized by internal disulfide bridges and have detrimental effects on their biological activity. In order to optimize exosomal yield, we explore two vesicle treatment protocols - dithiothreitol (DTT) and 3-[(3-cholamidopropyl)dimethylammonio]-1-propanesulfonic (CHAPS) - applied to the differential centrifugation protocol for exosomal vesicle isolation. The results show that CHAPS treatment does not affect vesicle morphology or exosomal marker distribution, thus eliminating most of THP interference. Moreover, the recovery and preservation of catalytic activity of two trans-membrane proteases, dipeptidyl peptidase IV and nephrilysin, was examined and found to be clearly superior after CHAPS treatment compared to DTT. Finally, proteomic profiling by mass spectrometry (MS) revealed that 76.2% of proteins recovered by CHAPS are common to those seen for DTT treatment, which illustrates underlining similarities between the two approaches. In conclusion, we provide a major improvement to currently-utilized urinary vesicle isolation strategies to allow recovery of urinary vesicles without the deleterious interference of abundant urinary proteins, while preserving typical protein folding and, consequently, the precious biological activity of urinary proteins which serve as valuable biomarkers.

## Introduction

Exosomes are nanovesicles (diameter 40–100 nm) actively released by most epithelial cells to the extracellular milieu *via* the endo-exosomal pathway by exocytosis of multivesicular bodies (MVB) [1], [2]. The discovery of exosome vesicles in urine [3] has rapidly opened new possibilities for the mechanistic understanding of biological processes and, importantly, has served as a source for novel biomarkers [4]. Consequently, exhaustive proteomic profiling of urinary exosomes has identified more than 1100 gene products, including 177 disease-related proteins derived from all nephron segments [5] and from the urogenital tract [6], [7]. The identification from urine of distinct exosomal transcription factors [8] and nucleic acids encoding proteins native to all nephron segments [9] is groundbreaking, and highlights the need to precisely understand their biology which plausibly reflects new aspects of disease pathways.

The aim of this study was to optimise the currently-available techniques and remove the abundant confounding urinary proteins which seriously interfere with the exosomal vesicle recovery, thus influencing the final yield and subsequent analytical power. The treatment of the exosomal pellet obtained by the serial centrifugation protocol with dithiothreitol (DTT) has previously been proposed as a solution to reduce such interference [5]. Furthermore, previous proteomic profiling studies have also identified a number of receptor proteins whose three-dimensional folding is stabilised by disulfide bridges. Accordingly, sortilin-related receptor, for example, has 33 predicted disulfide bridges and megalin has 159 predicted disulfide bridges [10], [11] fixing their respective molecular structures. Any study attempting to evaluate functions of exosomes should optimally follow an isolation protocol which preserves the correct folding and therefore, the functionality of the respective proteins. DTT is a strong reducing agent, however, and the exosomal proteins may accordingly be reduced and unable to refold properly upon reoxidation, hampering relevant functional studies leading to loss of biomarker promise.

Here, we hypothesized that interference of soluble proteins in the exosomal isolation process occurs due to aggregation, non-specific interactions as well as their gelling properties (as *e.g.,* for Tamm-Horsfall glycoprotein). Any chemical agent which solubilises these aggregates may also reduce contamination of the ultracentrifugation pellet by these proteins. For these benefits, we have used 3-[(3-cholamidopropyl)dimethylammonio]-1-propanesulfonic (CHAPS) as mild detergent which is known to solubilise THP [12] to largely exclude this interference. We then compared results after CHAPS treatment with those after the DTT treatment. Furthermore, we also compared the preservation of two key protease activities, including dipeptidyl peptidase IV (DPP IV) and nephrilysin (NEP), both previously shown to be associated with urinary exosomes [3], [5]. These two enzymes are stabilised by 5 and 6 disulfide bonds, respectively [13], [14]. Furthermore, we reveal for the first time the overlapping proteome subsets following CHAPS and DTT treatments. The proteomic profiling revealed that both CHAPS and DTT methods result in closely-related protein profiles, fully validating our method while CHAPS was found to be superior in maintaining functional protein integrity. Our method should greatly improve the exosome functional yield and, thus, possibilities to fully exploit the biomarker potential of exosomes.

## Results

### Vesicle Purification

**Figure 1:**
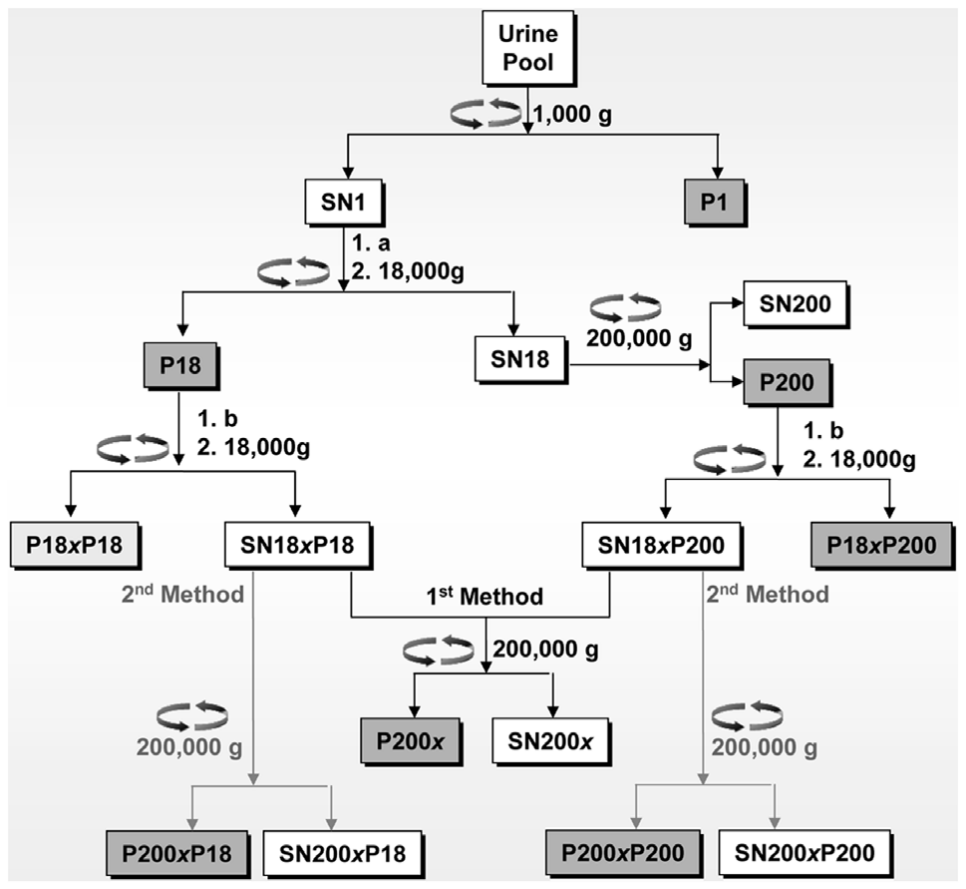
Urine vesicle enrichment workflow. **a.** Dialysis followed by vacuum concentration (approx.1∶25); **b.** either 1% CHAPS, ON at 4°C or 200 mg/mL DTT, 30 minutes at 37°C. The type of treatment is reflected in the sample name: the *x* is replaced by C in case of CHAPS treatment and by D in the case of DTT. SAMPLE NOMENCATURE: P: Pellet; SN: Supernatant; *x:* Detergent used for treatment b C: CHAPS, D: DTT. Generally, P/SN are followed by the shortened centrifugational speed (*e.g.*, 1 for 1,000 g), after detergent treatment C/D will be used as prefix of the fraction which underwent treatment. If double centrifugation has been performed, the sample name will reflect the chronological order of events with the last step first.

A schematic representation of the workflow used to isolated urine exosomal vesicles and abbreviations used for each product step is shown in Fig. 1. For the analysis, pooled urine samples were initially centrifuged at a relative centrifugal force (**RCF**) of 1,000 g for 20 minutes. The resultant supernatant (SN) was dialysed against deionized water and sample volume reduced to 1∶20 by vacuum concentration. The concentrated SN was centrifuged at 18,000 g and the resulting supernatant at 200,000 g. Both pellets (P18 and P200) were resuspended in a minimal volume of 200 mg/ml DTT or 1% (w/v) CHAPS and re-centrifuged again at 18,000 g. The two SNs were combined and further centrifuged at 200,000 g. Alternatively, CHAPS 18,000 g SNs were not pooled but centrifuged individually at 200,000 g to check THP sedimentation behaviour following CHAPS treatment.

### Transmission Electron Microscopy (TEM)

**Figure 2:**
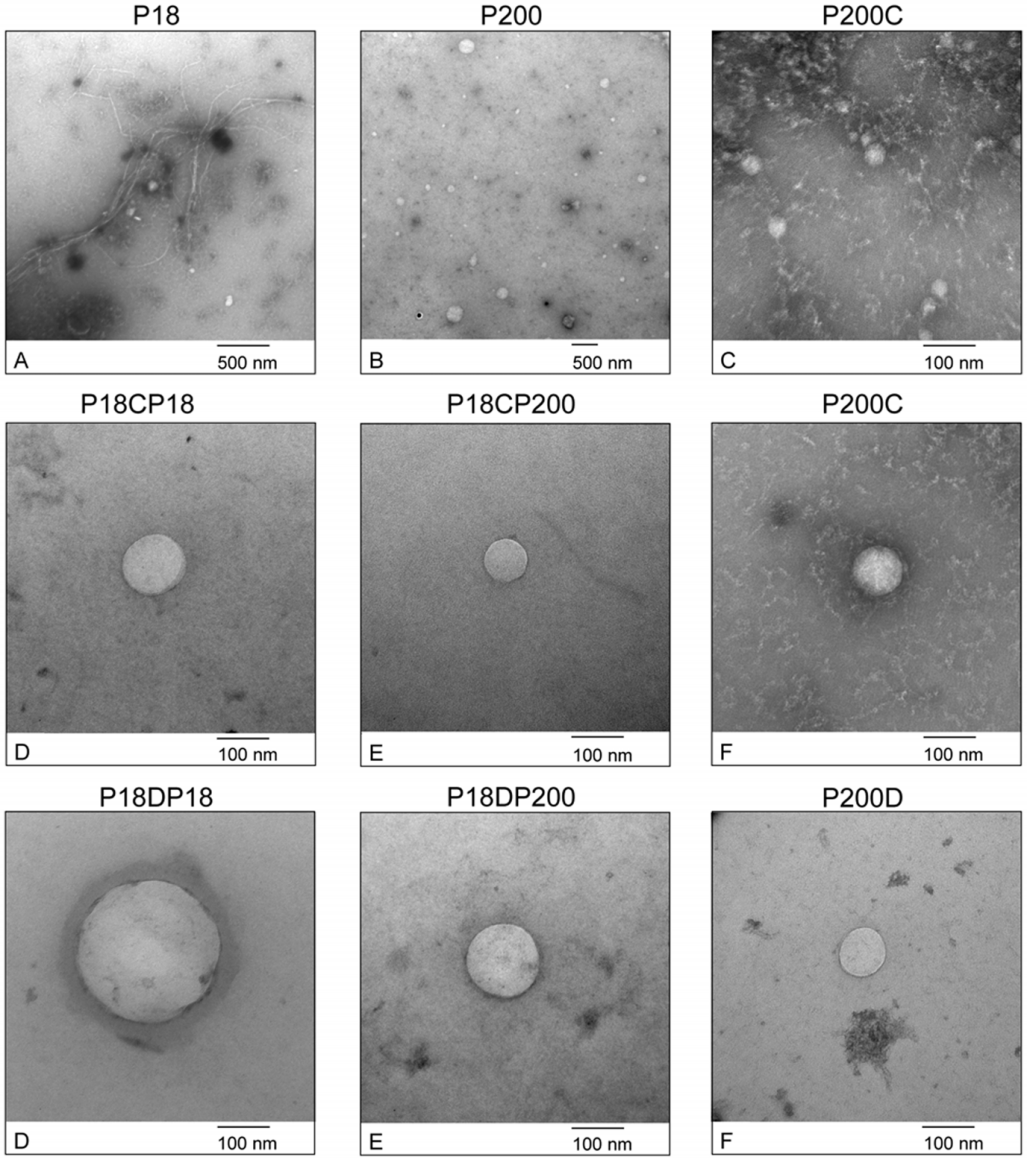
TEM analysis. Transmission electron micrographs of **P18** (Panel A) and **P200** (Panel B) at 10,000× and 5,000× magnifications, respectively. High-magnification (50,000×) of CHAPS- (Panels C-F) and DTT-treated (Panels G-I) vesicle preparations are represented.

Transmission electron microscopy was used to verify the presence and to evaluate qualitative features of the vesicles present in the crude preparation P18 and P200 (for abbreviations, see Fig. 1) and in CHAPS/DTT treated fractions (Fig. 2), as well as to assess the integrity of these vesicles following the respective treatments. As shown in Fig. 2A, the **P18** preparation contained a heterogeneous population of vesicles ranging in size from 50–100 nm in diameter. Furthermore, long polymeric filaments closely resembling THP polymers were detected along with associated vesicles [15], despite the fact that dialysis should remove factors favouring the tendency of THP to aggregate [16]. Crude **P200** (Fig. 2, Panel B) showed a heterogeneous population of vesicles whose size distribution (10–300 nm) and shape are consistent with those of exosomes and exosome-like vesicles previously described in urine [3], [17]. **TEM images** revealed that the preparations obtained after CHAPS or DTT treatments in *method 1* (see Material and Methods) yielded intact vesicles ranging from approximately 20 to 200 nm, dimensions which are characteristic for urinary exosomes [3], exosome-like [17] and apically-shed vesicles [18]. High magnification images (50,000×) after CHAPS (Fig. 2, panels C, D, E and F) and DTT treatments (Fig. 2, panels G, H and I) showed closely related morphologies and sizes. Another interesting observation was the detection of vesicles sized between 5 and 10 nm enriched in the P200C preparation, further highlighting the wide complexity, size distribution and heterogeneity of these fractions (Fig. 2, panel C).

### SDS-PAGE Analysis

**Figure 3:**
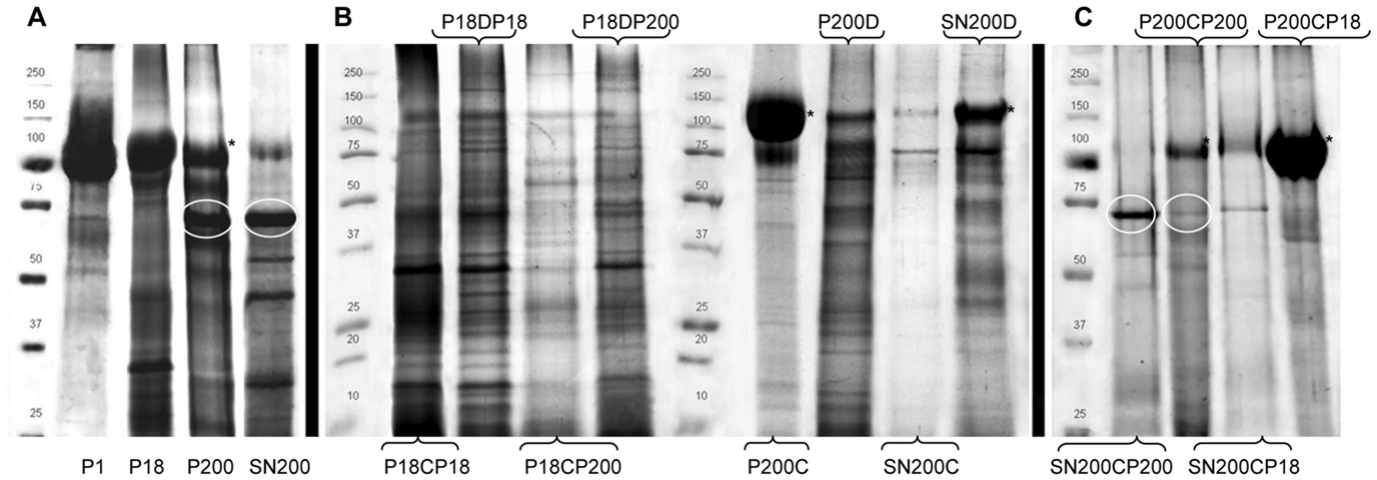
SDS-PAGE. **Panel A:** Gel Acrylamide T 12% constant. Fifteen µg of protein per lane of crude preparation **Panel B:** Gel Acrylamide T 8% constant. Ten µg of protein per fraction obtained in Method 1. **Panel C:** Gel Acrylamide T 12% constant. Ten µg of protein per fraction obtained in Method 2.

Figure 3 shows colloidal Coomassie-stained gels of crude fractions (Fig. 3, panel A), DTT fractions and CHAPS fractions obtained in method 1 (Fig. 3, Panel B) and CHAPS fractions obtained in method 2 (Fig. 3, panel C). The large band at around 100 kDa is the monomeric form of Tamm-Horsfall Protein, which abundantly sediments at a low-speed (1000 g) and then progressively, in decreasing amounts is recovered in the following centrifugation steps (**P18** and **P200**), with traces left in the final supernatant (**SN200**). After either DTT or CHAPS treatment of the crude **P18** and **P200** sediments, respectively, the pellet recovered again at a relatively low RCF (**P18CHAPSP18, P18DTTP18, P18CHAPSP200** and **P18DTTP200; for abbreviations, see** **Fig. 1**) showed only traces of THP. Surprisingly, after CHAPS treatment, in method 1 where the SNs were combined (Fig. 1), all the THP was recovered in the ultracentrifugation pellet (Fig. 3, Panel B **P200C**) instead of being in the SN (Fig. 3, Panel B **SN200C**) as was observed for DTT treatment (Fig. 3, Panel B **P200D** and **SN220D**). Interestingly, without pooling the SNs, THP present in the crude pellet (**P18**) after CHAPS treatment was not recovered anymore at 18,000 g, but at 200,000 g (Fig. 3, Panel C **P200CP18**). CHAPS treatment of crude P200 pellet without pooling gave a final sediment (Panel C, **P200CP200**) in which THP was present at the same amount with respect to the DTT pellet (Panel B **P200D**). Moreover, albumin (∼66 kDa), found in abundance in the crude pellet (Panel A, **P200**), was instead detectable in the supernatant after CHAPS treatment (Fig. 3, Panel C **SN200CP200**), with traces remaining in the 200,000 g pellet (Panel C, **P200CP200**).

### Profiling of Urinary Markers by Western Blotting

**Figure 4:**
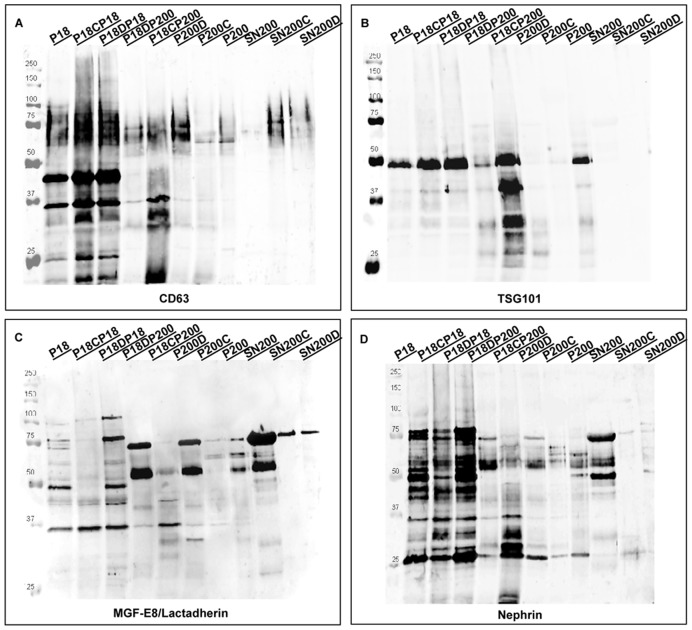
Western blotting analysis. Rabbit anti-CD63, Rabbit anti-TSG101, rabbit anti-MGF-E8/lactadherin and rabbit anti-nephrin [18], [50]. Ten µg of protein of fractions obtained in Method 1 were loaded on the gels.

Figure 4 shows the Western blotting of exosomal and podocyte markers.

### CD63

CD63 was abundantly found in **P18** and in the corresponding low-speed detergent-treated and DTT precipitates (Panel A; **P18CP18, P18DP18, P18CP200, P18D200**; for abbreviations, see Fig. 1). The CD63 isoform profiles exhibit a distinct distribution in these vesicle preparations, with sizes of approximately 65, 45 and 35 kDa, respectively, consistent with a heavy glycosylation pattern [19]. In treated high-speed pellets (**P200C** and **P200D**) and the crude **P200** fraction, CD63 was observed mainly at higher molecular weights (75 to 100 kDa). The broad band seen in Western blotting is most likely due to the high-degree of glycosylation by poly *N-*acetyl lactosamine, as reported by Engering et al. [20].

### TSG101

TSG101 was detected in the exosome fraction as a single band of approximately 46 kDa in **P18** and **P200** crude preparations but, significantly, after this fraction was treated (CHAPS or DTT), the antigen was recovered in the low-speed centrifugation preparations, namely **P18CP200** and **P18DP200**. In the latter two samples, in addition to the main band of approximately 46 kDa, other four lower molecular weight isoforms were also detected. It appears that the lower molecular weight bands are a specific feature of these two samples, especially of the CHAPS-treated fraction.

### LACTADHERIN/MFG-E8

In the P18 preparation, two main bands were visible with molecular weights of approximately 48 kDa and 35 kDa of lactadherin. Interestingly, after CHAPS treatment, only the 35 kDa band was detected (**P18CP18** and **P18CP200**). Notably, in the corresponding DTT pellet, (**P18DTTP18**), both the 46 and 35 kDa bands were present, in addition to two extra isoforms of approximately 77 and 100 kDa, specifically enriched in this fraction. The analysis of the crude **P200** preparation revealed three main bands whose molecular masses were approximately 53, 62 and 70 kDa. Interestingly, the 53 and 70 kDa forms were specifically enriched in both DTT preparations originating from the **P200** samples.

### NEPHRIN

A recent report has described the ability of glomerular podocyte microvilli to secrete non-exosomal vesicles by tip vesiculation [18]. This finding led us to explore this podocyte-specific marker of the slit-diaphragm in detail in the vesicle preparations, especially in the CHAPS or DTT-treated vesicles, in order to scout for specific functional features reflecting the critical elements of the glomerular filtration barrier. The whole array of samples was probed with an anti-nephrin antibody raised against the intra-cytoplasmic domain of the protein [21]. Surprisingly, the bulk of the nephrin-containing vesicles were recovered at low centrifugational speeds, (**P18CP18, P18DP18, P18CP200** and **P18DP200**). Interestingly, in the whole sample set, no full-length nephrin was observed, although different nephrin fragments were detected in the diverse preparations. This is in line with our previous results [22]. In the soluble fractions, nephrin was detectable only in the **SN170** fraction as 75, 65 and 48 kDa bands, while in the vesicle preparations, a wider range of bands was detected at 75, 65, 48 and 35 and 25 kDa.

### Dipeptidyl Peptidase IV (DDPIV) and Nephrilysin (NEP) Activity in CHAPS and DTT Fractions

**Figure 5:**
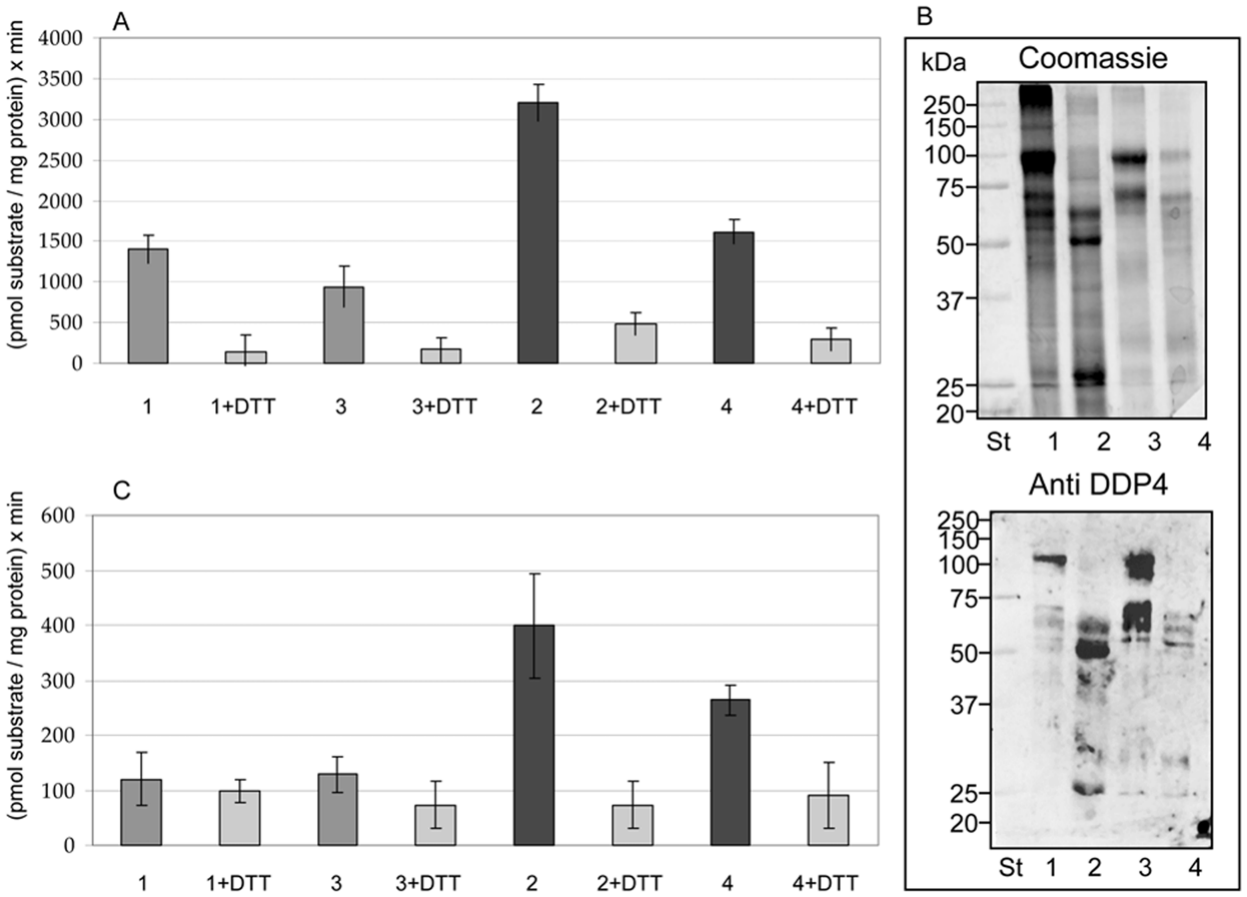
Proteases activity. Membrane-bound DPP IV (Panel A) and NEP (Panel C) peptidase activity profiles recorded in absence and presence of 10 mM DTT. Samples were dialysed at a MWCO of 300 kDa. DTT pellet 200,000 g (sample 1), DTT SN 200,000 g (sample 3), CHAPS pellet 200,000 g (sample 2) and CHAPS SN 200,000 g (sample 4) are represented. Columns compare DTT vs CHAPS after dialysis with a membrane of MWCO 300 kDa and in the presence of 5 mM DTT. Values represent mean ± SD of units of peptidase (UP) per milligram of protein per minute. Panel B represents the Coomassie gel and DDP immunodetection of the same samples. Ten µg of protein per fraction obtained in Method 1 were loaded on the gels after 300 kDa MWCO dialysis.

Figure 5 shows DPP IV and NEP activities measured in pellet and SN after CHAPS and DTT treatments. Before measuring the activity, samples were dialysed with a membrane with a molecular weight cut-off 300 kDa to remove DTT, CHAPS and potential soluble isoforms of these two proteases. Figure 5B shows Coomassie staining and the immunodetection of DPP IV after dialysis. In DTT 200,000 g pellets and SNs, DPP IV is visible at 110 kDa, while in CHAPS fractions two specific bands were detected whose molecular weights were approximately 52 and 25 kDa. Activity was recorded as pmol of product/min/mg protein (UP/mg protein). As shown in Fig. 5A, when compared, DPP IV activity in DTT and CHAPS fractions were similar despite the difference in the respective Western blotting pattern. Activity drastically decreased in the presence of DTT, suggesting that although disulfide bond reduction affects the activity, during dialysis DPP IV is able to regain its native conformation with a good recovery of its activity. For NEP, the activity decreased significantly (around 4-fold) in the DTT fractions when compared with the corresponding CHAPS fraction. This result shows that although DTT was removed by dialysis, there was a detrimental and irreversible effect of the reducing agent on the folding of NEP which led to a loss of the enzymatic activity. Once again, as a control, in the presence of DTT the activity decreased substantially in all the fractions. The activity measured in the SNs after dialysis with a molecular weight cut-off membrane of 300 kDa, along with CD63 signals detected in Western blotting (Fig. 4) indirectly points to the presence of nano-vesicles in these fractions, which were not collected in the pellet after the second ultracentrifugation.

### MS-based Identification of Proteins Released in the Supernatant after DTT and CHAPS Treatments

**Figure 6:**
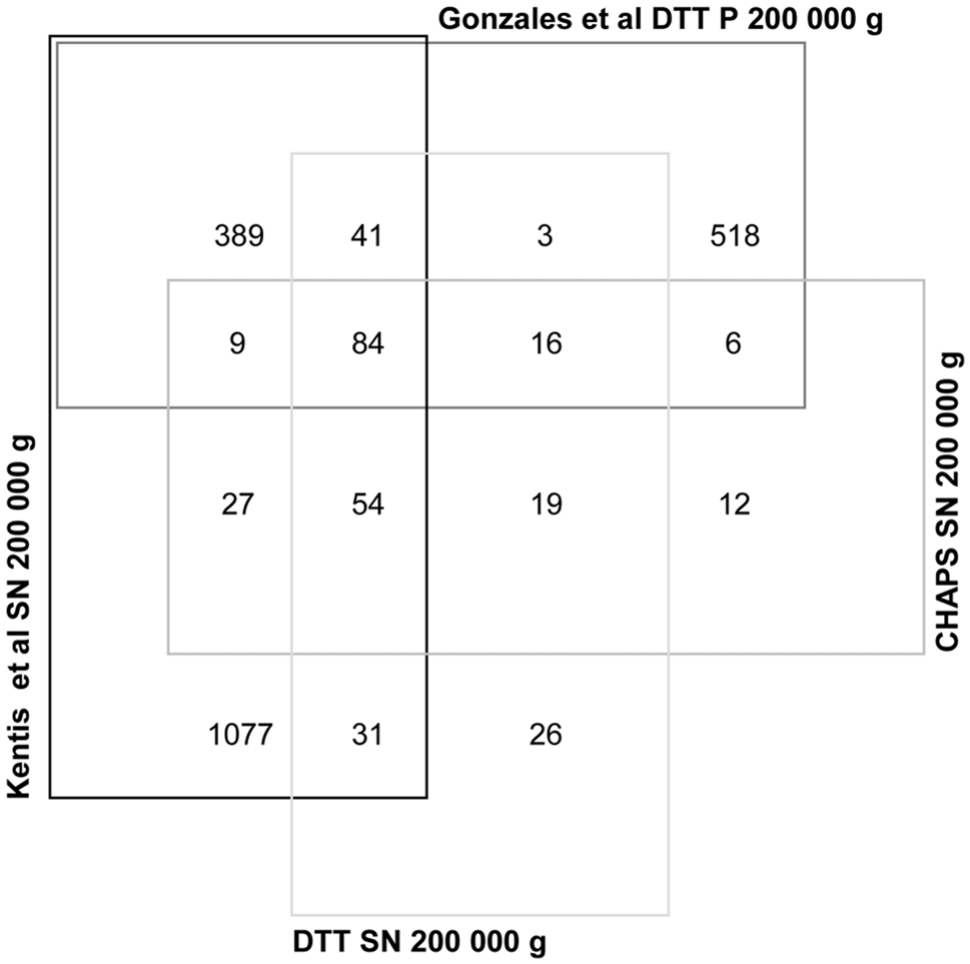
Protein identification comparisons. Venn diagram showing the distribution of the number of identified proteins presents in SN 200,000 g after CHAPS and DTT treatments. Protein identifications from the current study were compared to two other studies which were carried out using high-resolution mass spectrometers in gels on 200,000 g pellets after DTT treatment (Gonzales et al. 2008) [5] and 200,000 g supernatants (Kentsis et al. 2009) [23].

The supplemental material spreadsheet (Table S1) lists the proteins identified by the systematic MS analysis of the final 200,000 g supernatant after DTT (271 unique proteins) and CHAPS (243 unique proteins) treatments. For all the proteins identified, the uniqueness of the corresponding peptides was checked. Fig. 6 shows the analysis in comparison to the two most exhaustively-published data sets of proteins identified in the 200,000 g pellet [5] and SN [23] respectively. The Venn diagram shows that 76.2% of proteins found in the CHAPS fraction are common to the corresponding DTT fraction. This result confirms the close similarity between the two methods in respect to the preservation of major protein components. Interestingly, approximately 9.7% and 6.9% of protein in CHAPS and DTT supernatants, respectively, are consistent with the observations made by Gonzales [5], while 33.7% and 31% of proteins in CHAPS and DTT supernatants, respectively, are common to the Kentsis [23] data-set while 40.9% and 45.6% of the proteins in CHAPS and DTT SN, respectively, overlap with the two reference data sets. Finally, around 10% of proteins detected in our study are unique for both CHAPS and DTT supernatants.

Table 1 reports a selection of unique proteins not identified previously in urine either in 200,000 g pellets after DTT [5] treatment or in the 200,000 g SN [23] (the full list is reported in Table S1 and representative MS/MS spectrum of all those protein identified by 1 unique peptide is shown in Fig. S2). These unique identifications were compared with two of the most exhaustive studies on the whole urine [24], [25] along with ExoCarta database (http://exocarta.ludwig.edu.au/) [26] which provides a comprehensive content of proteins identified in Exosomes in multiple tissues and organisms. The bulk of identified proteins were found to be already annotated in the whole urine, but others like Ig gamma-4 chain, Ig gamma-3 chain C region and Ig alpha-2 chain have specifically been shown to associate with exosomes. Others, including Tumor Necrosis Factor receptor superfamily member 19L or palmitoyl-protein thioesterase 1 have all the potentiality to be part of the exosome proteome. These results suggest that in specific subpopulations of exosome vesicles, unique features can still be recovered in the SN after DTT and CHAPS treatments. This finding has practical consequences for their use. Figure S1 (supplemental material) reports the gene ontology distribution per protein class of identified proteins by the Panther classification system (www.panther.org) [27]. Once again the distribution of the protein classes is very similar in the 200,000 g CHAPS and DTT supernatants.

**Table 1:** Protein identification in DTT and CHAPS supernatant.

Fraction	Swiss-Prot accession number	Coverage %	aUnique Peptides	Mascot score	Descriptions	Exocarta	Adachi	Marimuthu
CHAPS	O95497	10.33	3/4	180.32	Pantetheinase GN = VNN1		Y	Y
CHAPS	P50897	9.15	2/2	97.96	Palmitoyl-protein thioesterase 1 GN = PPT			Y
CHAPS	P01040	30.61	2/2	109.9	Cystatin-A GN = CSTA			Y
CHAPS	Q86SR0	45.36	2/2	114.84	Secreted Ly-6/uPAR-related protein 2 GN = SLURP2			
CHAPS	Q969Z4	2.56	2/2	109.04	Tumor necrosis factor receptor superfamily member 19L GN = RELT		Y	Y
CHAPS	P61916	10.60	1/1	66.37	Epididymal secretory protein E1 GN = NPC2		Y	Y
CHAPS/DTT	P01861	14.98 and18.35	1/5 and 0/7	217.67 and 354.82	Ig gamma-4 chain C region GN = IGHG4	Y		
CHAPS/DTT	P01877	14.41 and 28.82	1/4 and 3/7	360.53 and 384.08	Ig alpha-2 chain GN = IGHA2	Y		
CHAPS/DTT	P37235	6.22 and 4.15	1/1 and 1/1	81.02 and 58.38	Hippocalcin-like protein 1 GN = HPCAL1	Y		Y
CHAPS/DTT	P15289	8.09 and 14.00	2/2 and 5/5	123.11 and 293.32	Arylsulfatase A GN = ARSA			Y
CHAPS/DTT	P41222	24.74 and 21.05	6/6 and 5/5	304.59 and 309.59	Prostaglandin-H2 D-isomerase GN = PTGDS		Y	Y
CHAPS/DTT	Q14508	38.71 and 11.29	4/4 and 2/2	216.44 and 103.89	WAP four-disulfide core domain protein 2 GN = WFDC2		Y	Y
CHAPS/DTT	P31949	15.24 and 15.24	1/1 and 1/1	80.02 and 92.18	Protein S100-A11 GN = S100A11	Y	Y	Y
DTT	P02788	6.48	5/5	227.66	Lactotransferrin GN = LTF	Y	Y	Y
DTT	P19835	14.61	7/7	339.62	Bile salt-activated lipase GN = CEL	Y		Y
DTT	Q9Y646	16.95	6/6	334.27	Plasma glutamate carboxypeptidase GN = PGCP			Y
DTT	P55259	4.28	2/2	136.92	Pancreatic secretory granule membrane major glycoprotein GP2 GN = GP2			Y
DTT	P01860	13.79	0/5	256.39	Ig gamma-3 chain C region GN = IGHG3	Y		

Partial list of proteins not previously reported in urinary exosomes and in 200,000 g supernatants.

a Unique peptides on the total number of peptides.

## Discussion

Exosomes found in all biological fluids, including the urine, are emerging as a novel class of cell products plausibly changing our views of cell behaviour and processes like intercellular communication [28]. While an increasing variety of exosome constituents including specific proteins, DNA and RNA species are being identified, little is still understood of their precise biological roles. Likewise, the method optimisation for exosomal isolation and interactions to appreciate their distinct roles in biology remain to be characterised in detail. The aim of this study was to optimize methods which increase the recovery of urinary exosomal vesicles and optimise the preservation for functionality without notable interference with inherent soluble protein constituents of urine.

Tamm-Horsfall Protein is the most abundant glycoprotein normally found in urine [29]. It is expressed abundantly in the thick ascending limb of the loop of Henle and the early distal convoluted tubule [30]. THP contains the most varied array of associated glycans of any human glycoprotein, which suggests a capacity for adhesion to a variety of ligands [31]. In the urine, THP may precipitate due to many factors of the immediate physico-chemical microenvironment and THP, accordingly, is the main constituent of hyaline urinary casts [32], [33]. More recently, Fernández-Llama and colleagues [15] demonstrated that abundant exosome vesicles are entrapped within urinary THP polymers. In order to overcome such a confounding interference in the analysis of the total exosome proteome, a strong denaturation of its 24-disulfide bridges by DTT have been proposed [5], [15]. This leads to unfolding of the zona pellucida (ZP) domain responsible for protein polymerization [34]. Alternatively, ultracentrifugation methods employing sucrose gradient or cushion in deuterated buffer to remove THP have been proposed [7], [17].

We introduce here distinct modifications, as summarised in Fig. 1, to the well-established method of exosome isolation which is based on serial differential centrifugations [34]. Firstly, a very low-speed centrifugation at 1,000 g was introduced to remove abundant cells, cell debris, nuclei, bacteria and the bulk of THP interfering by entrapping the exosomes. As evident by SDS-PAGE analysis of this fraction, the approach was efficient (Fig. 3). Although we cannot fully appreciate the fact that a fraction of vesicles retained at this point may have been entrapped within THP polymers, the introduction of this low-speed centrifugation step avoided a potential contamination resulting from intracellular vesicle release as a result of cytolysis following freeze-thaw cycles and hypotonic shock during dialysis. Moreover, vesicles can also be shed from cells during the isolation process [35]. Thus at the end of it is unclear whether vesicles are present in urine at the collection time or if are they created from cells present in the sample during the isolation process. Furthermore, a dialysis step was introduced to decrease the salt concentration in order to favour the de-polymerization of THP and avoid its precipitation during the following centrifugation steps. Nonetheless, THP fibrils were still present in the low-speed centrifugation pellet, as shown in Fig. 2, possibly due to the increased THP concentration following the reduction of the starting volume by vacuum concentration. This step was introduced with the double aim of handling a large amount of urine in a small volume and, secondly, to mimic a proteinuric condition and, thus, to evaluate the extent of albumin interference which is well-represented in the first crude pellets along with THP. To overcome these barriers, our protocol was modified particularly to facilitate the release of entrapped vesicles from THP polymers, removing at the same time the interference of soluble proteins like albumin.

After systematic trials, we selected two approaches to manage THP interference. The first involved the well-established DTT treatment [3], [5], [15], [34] which unfolds the zona pellucida (ZP) domain responsible for protein polymerization [36]. The second method involved the addition of a mild detergent which has previously been shown to solubilise THP efficiently [12]. CHAPS, a non-denaturating zwitterionic detergent is ideally suited for the disruption of non-specific protein interactions, while also protecting the conformation of the protein(s) of interest [37]. The bulk of THP and albumin were successfully removed from the exosomal pellet and after the treatment with CHAPS, were found in the supernatant as shown in Fig. 3. CHAPS is known to break protein-protein interactions [37], [38]. However, depending on the microenvironment, the strongest protein-protein interactions, such as those found in the tetraspanin web [39], are preserved.

When analysed by electron microscopy, pellets retained after both low and high-speed centrifugations treated with CHAPS and/or DTT showed a characteristic spherical morphology limited by a bilipidic layer (Fig. 2) and with diameters ranging from between 30 to 150 nm. These characteristics are consistent with the observed size and morphology of vesicles previously described in urine [3], [5], [17], [18], [34], [40]–[42]. Indeed, it is clear that urinary vesicles represent a tremendous heterogenity in size and in the exosomes, marker patterns are evident as shown in the Western blot analysis. The concomitant presence of other specific markers like nephrin and lactadherin complicate the semantic definition of such urinary fractions which can include what is generally called microparticles, ectosomes, exosomes, exosome-like vesicles and shed vesicles. Furthermore, these results clearly suggest that the whole vesicle is a structure resistant to treatment with a detergent such as CHAPS, which in turn demonstrates that this may be used for vesicle isolation without having a deleterious effect on the structural conformation of vesicles. In our analysis, after an ON incubation in 1% CHAPS, which is able to solubilise a detergent-resistant domain almost completely within one hour of incubation [43], we were able to perform comparative analysis of CHAPS- and DTT-treated vesicle populations by TEM (Fig. 2). This suggests that urinary vesicles are detergent (CHAPS)-resistant. Furthermore, the MS-based proteomic profiling led to the identification of 247 unique proteins in CHAPS SN and 274 in DTT SN, respectively. This finding indirectly confirms the resistance of these vesicles to detergent solubilisation. The Venn diagram in Fig. 6 shows that around 75% of identifications in CHAPS or DTT are shared. Although there maybe still differences due to the different composition of the solution of the second ultracentrifugation step, which happened in presence of 200 mg/ml DTT and 1% (w/v) CHAPS respectively, 75% of homology in terms of protein identification clearly highlights the detergent-resistant features of urinary nanovesicles. In fact, if CHAPS had lysed and solubilised the vesicle contents, it would be expected to find a protein data set which would be highly biased toward the exosomal proteome. Furthermore, the distribution of the protein classes by Panther classification system (Fig. S1) was similar between CHAPS and DTT in respect to several exosomal proteins. Comparison of our results with the published proteomic data on exosomes and exosome free supernatants [5], [23] showed that more than 50% -exosomes pellet- and 70% -SN exosome free- of the identified proteins were shared, respectively. Interestingly, around 40% of identified proteins are common to the 4 data sets. In spite of differences in the methodological approach, the off-gel utilized in this study with respect to the in-gel method utilized in the comparative protein data set [5], [23], instrumentation and bioinformatics tools, the 3 data sets showed high concordance in the identifications since the stringency of MS identification criteria used (described in detail in the original publications on each dataset) was reasonably high in each case. Therefore, false-positive identifications should represent less than 1% of the entries. Keeping in mind that these vesicles were found to be resistant to harsh treatments, this evidence shows an incomplete recovery of vesicles by the classical ultracentrifugation protocols which may lead to a preferential enrichment of vesicular subpopulations which plainly share a common set of proteins (exosomal markers) along with some specific ones (inherent to a possible sub-population). This is of significant practical importance as the increased yield of vesicles isolated and the confidence in the MS identification and quantification of low-abundant proteins from vesicle pellets could be of crucial importance to identify biomarkers represented in very low amounts. Currently, modern proteomics aim for the analysis of sub-cellular proteomes for markers discovery. In this regard, two special kinds of extracellular vesicles such as exosomes and membrane plasma shedding vesicles are emerging as excellent biological sources to be applied in the discovery of non-invasive organ-specific disease biomarkers.

Protease activity of dipeptidyl peptidase (DPP IV) and nephrilysin (NEP) showed that the CHAPS method is a useful alternative to DTT when full preservation of biological activity of all the disulfide bridge containing proteins is needed. Particularly, NEP structure consists of a short N-terminal cytoplasmic domain, followed by a single trans-membrane helix, and a large C-terminal extracellular domain that contains the active site [44], [45]. The extracellular domain of NEP contains 12 cysteine residues, all components of six disulfide bridges. Four of these are located within the catalytic domain; a single disulfide bridge is found within the inter-domain linker fragments, and one is present within domain 2. All of these participate in maintaining the structure consisting of two multiply-connected folding domains which embrace a large central cavity containing the active site [46]. Evaluation of NEP proteolytic activity after reduction and re-oxidation (DTT treatment and subsequent removal of DTT) of disulfide bridges highlights that this may result in a severe misfolding, leading to an impairment of NEP activity. As shown by our results, NEP activity is better preserved in the CHAPS protocol.

Western blotting of CHAPS and DTT fractions revealed a very similar pattern. Firstly, after both treatments, the bulk of the tetraspanin family exosomal markers associated with late multivesicular endosomes CD63 [47] was recovered at a low centrifugal speed with a broad distribution of isoforms. During urine formation, the final product for voiding contains vesicles originating from a wide range of different cellular origins and hence, it was not a surprise to find such a heterogeneity which is a reflection of specific cell-sorting and trafficking pathways, but also of distinct origin upstream in the podocyte by finding fractions of nephrin in exosomes. Finally, although protease degradation cannot be excluded, at the time of its occurrence this activity seemed to be very specific and related to all those fractions which retained at a low-speed centrifugation. In support of this evidence, the immunodetection of cytosolic TSG-101 showed a fragmentation pattern in only one fraction **P18CP200** which was not seen in other fractions where the signal was detected in equal amounts (Fig. 4). Furthermore, the ability to detect the intra-vesicular localised marker TSG101 [48]–[50] underlines retained integrity of the vesicles during the whole purification methodology.

Lactadherin (MFG-E8 or SED1) is known to participate in a wide variety of cellular interactions [51] and has previously been shown to be released in association with exosomes [52]. An interaction has been reported between the discoidin/C domains and the pellucida zone [53] and one could speculate about a possible interaction with THP (ZP domain). Finally, lactadherin isoforms have been previously identified which are reflections of physiological state and distinct cell type origins [54], [55]. MFG-E8 immunodetection (Fig. 4) reveals a wide distribution of bands with specific signatures for each pellet. Proteomic profiling [3], [5] has identified potential interacting partners of MFGE-E8 (such as β-integrin) in exosomes. On the other hand, shed vesicles and apoptotic bodies have phosphatidyl-serine on the surface and MFG-E8 could mediate interactions between these vesicles and exosomes, and as well as THP. However, this remains to be fully established.

Surprisingly, immunodetection with anti-nephrin revealed a comparable distribution of fragments to those seen in nephrinuria patients [56]. Looking at the Western blot molecular weights and the intracellular location of the specific epitope recognised by our antibody, we propose that this vesicle preparation contains membrane-associated nephrin fragments. Whether these fragments were orientated right-side-out or inside-out has to be confirmed by further analyses. Identification of shed podocytes in urine of healthy subjects and patients affected by glomerular diseases has also been proposed previously [57], [58]. More recently, it has been reported that small podocalyxin-positive vesicles which are negative for exosomal markers originate from microvilli of podocytes in both healthy samples and those from patients with glomerular diseases [18]. Detection of nephrin and exosomal markers in the same fraction may suggest a protein turnover through clathrin- or raft-mediated endocytosis [54]. After internalisation by either mechanism, vesicles are sorted into the endosomal pathway which could redirect vesicles to be either degraded or secreted as exosomes. The minute amount of nephrin excreted into urine of healthy subjects could reflect a physiological turnover of the slit diaphragm. In support to this, nephrin has been detected by immune electron microscopy not solely at the slit diaphragm area but also along lateral podocyte membranes and, eventually, in the urine [56]. The detection of the same nephrin fragmentation pattern in the whole urine of diabetic nephropathic patients [56] suggests that an increase in this dynamic process may accurately reflect worsening podocyte injury. A better understanding of the mechanism(s) which accelerate the turnover of this key structure of the glomerular filtration barrier may provide a critical new insight into the identification of early markers of glomerular impairment during disease progression.

In conclusion, an improved method was developed to exclude most of the interference of soluble proteins present in urinary vesicle isolation. CHAPS treatment appears superior in preserving the activity of constituent proteins of membrane vesicles while simultaneously removing interference of soluble proteins (THP, albumin). Thus, our method offers a new protocol to prepare urinary vesicles for –omics, analytical or functional studies.

## Materials and Methods

### Urine Collection

Urine samples were collected from ten non-smoking healthy laboratory volunteers (5 female and 5 male) whose ages ranged from between 20 to 40 years. Donors verbally assented the donation of their own urine. Samples were not named and after being anonymously tested by Combur 10 Test®D dipstick (Roche Diagnostics; Mannheim, Germany), specimens were pooled together. There was no history of renal dysfunction in any of the subjects or drug administration during sample collection. Moreover, Combur test was negative for all samples. Hence, we implied that ethical approval was not necessary.

The first morning urine was processed within 3 hours of collection. At collection, the first 50 mls were discarded and the remaining void urine was collected for analysis.

### Vesicle Purification

A schematic representation of the methodology used to isolate nanovesicles is shown in Fig. 1. In summary, pooled urine samples were initially centrifuged at a relative centrifugal force (**RCF**) of 1,000 g for 20 minutes. The resultant supernatant (SN1) was retained and split into aliquots of 500 mL. Upon thawing, these samples showed cryo-precipitate, whose complete dissolution was carried out by vortexing the sample at room temperature (RT) until complete the solution became clear. Next, 500 ml aliquots of SN1 were dialysed at 4°C against deionised water (3 changes, 10 L each over 24 hours) and the volume was subsequently reduced up to 20 mL using vacuum concentration (miVac, GeneVac, Suffolk, Ipswich, UK). The concentrated SN1 was centrifuged (Avanti®J-26 XP centrifuge, Beckman Coulter, Fullerton, CA, USA) at 15,000 revolutions per minute (rpm) (∼18,000 g) for 30 minutes at RT in a fixed angle rotor (Beckman JA-20, Fullerton, CA). The 18,000 g SN (**SN18**) fraction was subjected to an ultracentrifugation step using the OptimaTM L-90 K preparative ultracentrifuge (Beckman Coulter) at 44,000 rpm (∼200,000 g) for 2 hours at RT in a fixed-angle rotor (Beckman 70Ti, Beckman). The pellet (P200) was resuspended in 3 mls of deionized water. Aliquots (2 mg of total protein) of resolubilised crude P18 and P200 were treated with either 1% (w/v) of 3-[(3-cholamidopropyl)dimethylammonio]-1-propanesulfonic acid (CHAPS) overnight (ON) with end-over-end agitation at 4°C (5 ml final volume) or with 200 mg/ml of dithiothreitol (DTT), independently of one another, for 30 minutes at 37°C in accordance with the recommendations of Fernández-Llama et al [15] with vortexing every five minutes. Here, a final volume of 5 mls was also used. These two solutions (*i.e.* P18 and P200, CHAPS and DTT treated pellet) were subjected to a second series of centrifugations at 15,000 rpm followed by an ultracentrifugation at 44,000 rpm, as shown in Fig. 1. After the first centrifugation at low-speed (15,000 rpm, which corresponds to a RCF of 26,000 g in the Beckman JA-20 rotor for a 5 mL solution), the SNs from same treatment were pooled together (10 ml final volume) and centrifuged at 44,000 rpm (corresponding approximately to an average RCF of 180,000±20,000 g in the fixed angle rotor, Beckman 70Ti).

For method 2, 0.5 mg of crude protein obtained from P18 and P200, respectively, was treated with CHAPS as in method 1 without pooling the SN after the first low-speed pellet (15,000 rpm). Instead, they were subjected individually to an ultracentrifugation step at 44,000 rpm, which corresponds approximately to an average RCF of 190,000±10,000 g in the fixed angle rotor selected for use (Beckman 70Ti, Beckman Coulter) using a sample volume of 5 ml.

### Determination of DDP IV and NEP Catalytic Activity

DPP IV activity was measured in triplicate by using HGly- Pro-β-naphthylamide as substrate, following the method of Liu and Hansen [59]. The NEP assay was carried out by incubating samples with N-dansyl-D-Ala-Gly-pNOH2-Phe-Gly ([D]AG (pN)PG, a dansyl derivative), following the protocol of Florentin et al [60].

### Electrophoresis, Western Blot, Electron Microscopy and MS Analysis

A full description of the methods is provided in supplementary material information (Material and Methods S1).

## Supporting Information

Figure S1 **Classification of proteins in the supernatant 200,000 g after DTT and CHAPS treatment.** The 2 sets of identified proteins were classified to their gene ontology groupings using the PANTHER classification system (www.pantherdb.org).
(TIF)

Figure S2 **Representative MS/MS spectra of peptides from selected proteins in Table 1that were identified based on single peptide evidence.** (A) Protein S100-A11, (B) Hippocalcin-like protein, (C) Epididymal secretory protein E1.
(TIF)

Table S1 **List of proteins identified by the systematic MS analysis and classified by PANTHER classification systemof the final 200,000 g supernatant after DTT (271 unique proteins) and CHAPS (243 unique proteins) treatments.**
(XLS)

Material and Methods S1 **More detailed description of: protein quantification, SDS-PAGE, western blotting, negative transmission electron microscopy, LC-MS/MS analysis and data analysis.**
(DOC)
