# Supplementary figures and images for: Biochemical and Physical Characterisation of Urinary Nanovesicles following CHAPS Treatment
Source: PLoS One. 2012 Jul 12;7(7):e37279. doi: 10.1371/journal.pone.0037279 (PMC3395701; doi:10.1371/journal.pone.0037279)

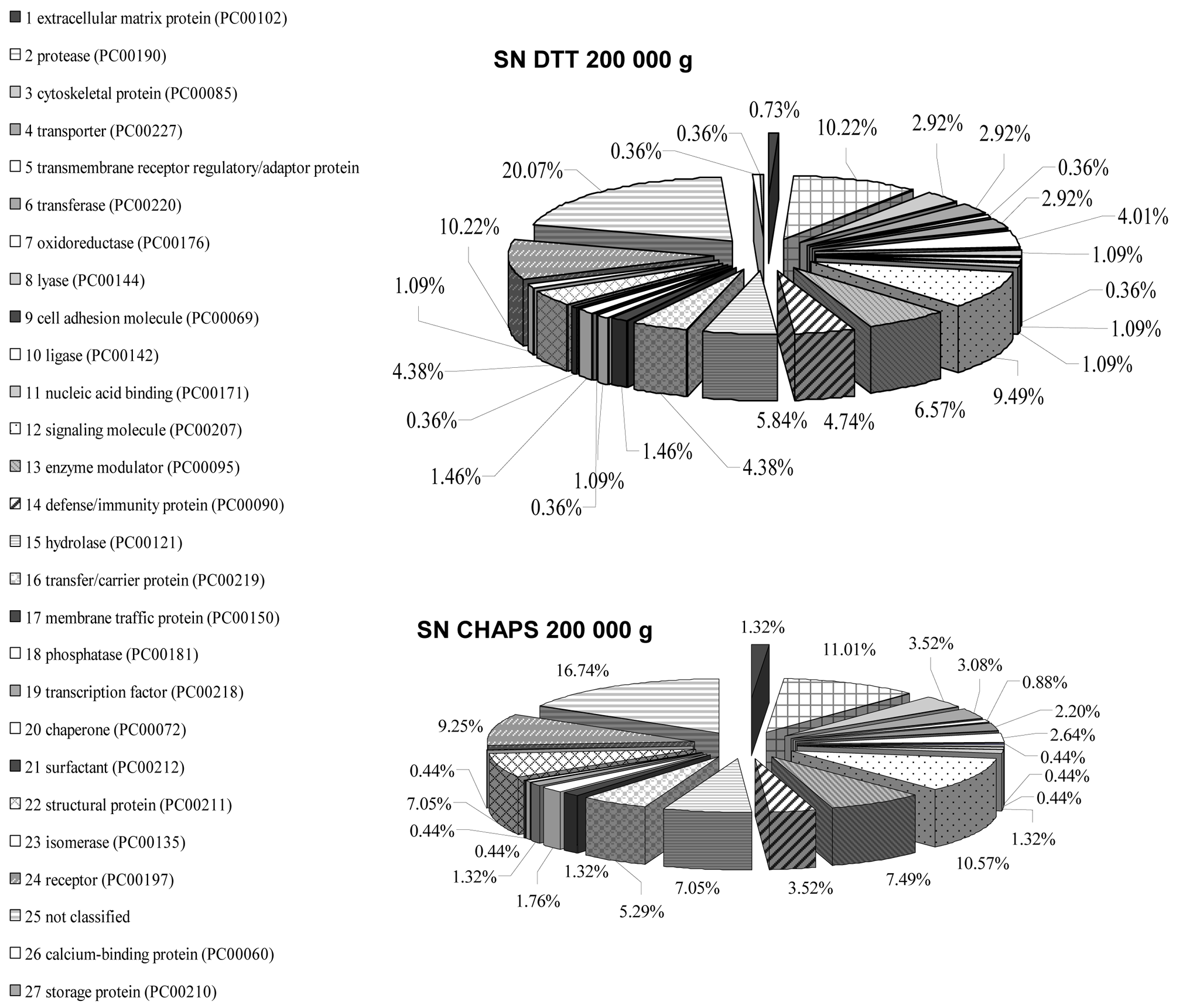

Supplement: Figure S1 — Classification of proteins in the supernatant 200,000 g after DTT and CHAPS treatment. The 2 sets of identified proteins were classified to their gene ontology groupings using the PANTHER classification system (www.pantherdb.org). (TIF) [file pone.0037279.s001.tif]

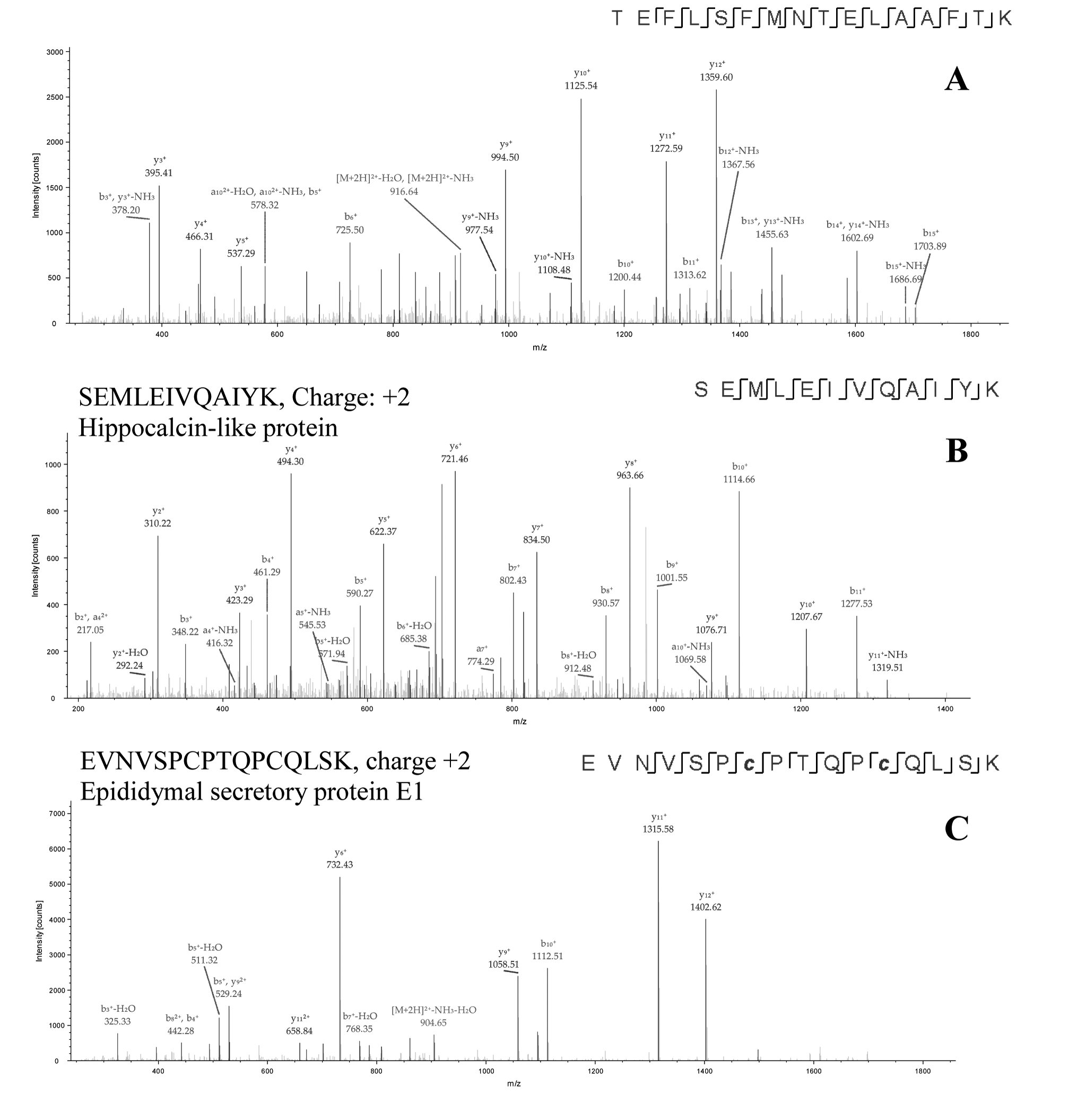

Supplement: Figure S2 — Representative MS/MS spectra of peptides from selected proteins in Table 1that were identified based on single peptide evidence. (A) Protein S100-A11, (B) Hippocalcin-like protein, (C) Epididymal secretory protein E1. (TIF) [file pone.0037279.s002.tif]
