# Supplementary material for: Biochemical and Physical Characterisation of Urinary Nanovesicles following CHAPS Treatment
Source: PLoS One. 2012 Jul 12;7(7):e37279. doi: 10.1371/journal.pone.0037279 (PMC3395701; doi:10.1371/journal.pone.0037279)
Supplement: Material and Methods S1 — More detailed description of: protein quantification, SDS-PAGE, western blotting, negative transmission electron microscopy, LC-MS/MS analysis and data analysis. (DOC) [file pone.0037279.s004.doc]

**Supplemental Material**

**Protein quantification, SDS-PAGE and Western Blotting**

The protein concentration in all urine fractions were measured by Coomassie Protein Assay [1]. SDS-PAGE was carried out according to the recommendations of Laemmli [2]. Gels were either stained by homemade colloidal Coomassie blue staining [3] or transferred to a nitrocellulose membrane [4]. Membranes were blocked overnight (ON) at room temperature (RT) with the Odyssey blocking buffer solution (LI-COR Biosciences, Lincoln, NE, USA). All incubation steps with antibodies were performed in a 1:1 (v/v) mixture of Odyssey blocking buffer and PBS with 0.1 % (v/v) Tween 20 (PBST). Antibodies were diluted in accordance with supplier’s guidelines for 2 hours at room temperature. Rabbit antiCD63 (H193) sc15363 (Santa Cruz), dilution 1:500 (v/v); rabbit anti TSG101 (Sigma) 0.5 μg/mL dilution; rabbit antiMFG-E8/lactadherin (H60); sc-33545v (Santa Cruz) 1:500 (v/v) dilution; rabbit antinephrin (in house) 1:500 (v/v). Secondary antibody goat antirabbit λ 800 (LI-COR Biosciences) was diluted 1:5000 (v/v) in PBS tween 0.1 % (v/v) plus 0.01 % (w/v) SDS for 2 hours at room temperature. Images were acquired by Odyssey Infrared Laser Scanner (LI-COR Biosciences, Lincoln, NE, USA)

**Negative Transmission Electron Microscopy**

50 μg of vesicle preparations were fixed with 1% (v/v) glutaraldehyde (Sigma Aldrich) in water. Fixed vesicle preparations were spotted onto a Formvar/Carbon 300 mesh grid (Agar Scientific, Stansted, UK) and dried at RT. The grids were washed twice in 0.1 M PBS and incubated in 1% (w/v) OsO_4_ in 0.1 M PBS for 30 min on ice. After five 5 min washes (3 with PBS and 2 with water), exosomes were stained with 5% (w/v) uranyl acetate in water for 10 min [5]. After staining, vesicle populations were monitored by JEM-2100 transmission electron microscopy (Jeol Ltd, Tokyo, Japan).

**LC-MS/MS analysis**

100 μg of protein were reduced by 10 mM tris(2-carboxyethyl)phosphine (TCEP) in 100 mM Tris–HCl pH 8.8 , 8 M Urea, 0.1 mM EDTA and 1 % (w/v) sodium deoxycholate (DOC) for 1.5 h at Room Temperature (RT) in the dark. Alkylation was carried out by 20 mM iodoacetamide (IAA) in 100 mM Tris–HCl pH 8.8, 0.1 mM EDTA for 1.5 h at RT in the dark. Excess of IAA was quenched by 20 mM N-acetyl cysteine in 100 mM Tris–HCl pH 8.8 , 0.1 mM EDTA for 0.5 h at RT in the dark. Before trypsin digestion reduced and alkylated samples were concentrated/delipidated by chloroform/methanol precipitation according Wessel and Fugge [6]. Briefly, to 150 μL of sample solution were added first 400 μL of 100% MeOH; and then 200 μL of Chloroform after having vortex well 400 μL of mQ water was added and then spun at 5,000 g for 5 minutes at RT. The upper layer was removed and 600 μL of 100% MeOH was added to the tube. Finally, protein was recovered by centrifugation at max of the speed for 30 minutes and the pellet dry by speed vac. Digestion was carried out using trypsin (modified sequencing grade; Promega, Madison, WI) at 37°C for 16 h in 50 mM Tris-HCL pH 8.0 and 1 % DOC which is compatible with trypsin activity [7]. The digested solutions were acidified with 1 % (v/v) formic acid (up to around pH 2) and centrifuged at max of the speed for 20 minutes. The supernatant was collected and the pellet were washed for with 1 % (v/v) formic acid and sonicated for 5 minutes and centrifuged at max of the speed for 20 minutes. This was repeated twice [8]. All the SNs were pooled together and were then desalted and concentrated by using reverse phase cartridges Sep-Pak tC18 according to manufacture’s instructions according to the manufacturer's instructions (Waters, Mississauga, ON). Briefly, the columns were conditioned and equilibrated with 1 mL 100 % methanol and 1 mL 80 % (v/v) acetonitrile (ACN), respectively and successively washed with 4 ml of 0.1 % (v/v) formic acid. The sample solution was then loaded onto the column, and the columns were washed with 6 mL 4 mL of 0.1 % (v/v) formic acid. The tryptic digestions were eluted with 1 mL 80% (v/v) ACN, 0.1% (v/v) formic acid and lyophilized (Thermo). Lyophilized samples were rehydrated with 0.1% (v/v) formic acid to give 1 μg/μL concentration of the equivalent starting protein amount.

Nanoflow electrospray ionization tandem mass spectrometric analysis of peptide samples was carried out using LTQ-Orbitrap Velos (Thermo Scientific, Bremen, Germany) interfaced with the Agilent 1200 Series nanoflow LC system. The chromatographic capillary columns were used with a flow rate of 300 nL per minute. The peptides were eluted using a linear gradient of 7-30% acetonitrile over 50 min. Mass spectrometry analysis was carried out in a data-dependent manner with full scans acquired using the Orbitrap mass analyzer at a mass resolution of 60,000 at 400 *m/z*. For each cycle, the twenty most intense precursor ions from a survey scan were selected for MS/MS and detected at a mass resolution of 15 000 at *m/z* 400. The fragmentation was carried out using higher-energy collision dissociation as the activation method, with 40% normalized collision energy. The ions selected for fragmentation were excluded for 30 seconds. The automatic gain control for full FT MS was set to 1 million ions and for FT MS/MS was set to 0.1 million ions with a maximum time of accumulation of 750 and 100 ms, respectively.

**Data Analysis**

The mass spectrometry data was processed using Proteome Discoverer (Version 1.2.0.208) software (Thermo Fisher Scientific) and searched using Mascot. The search parameters used were: oxidation of methionine, deamidation at N and Q, protein N-terminal acetylation, N-pyroglutamate for N-terminal Q/N and carbamidomethylation of cysteine residues as variable modifications. A maximum of one missed cleavage was allowed for tryptic peptides with a minimum length of 7 amino acids. The peptide and protein data were extracted using high peptide confidence and top one peptide rank filters. False discovery rate was calculated by enabling the peptide sequence analysis using the decoy database. Mass error window of 20 ppm and 0.1 Da were allowed for MS and MS/MS, respectively. 1% FDR was used as a cut-off value for reporting identified peptides. In addition to the target-decoy approach, for all those proteins identified, the uniqueness of the corresponding peptides was carried out.

1. Bradford MM (1976). A rapid and sensitive method for the quantitation of microgram quantities of protein utilizing the principle of protein-dye binding. Anal Biochem 72: 248–254.
2. Laemmli UK (1970) Cleavage of structural proteins during the assembly of the head of bacteriophage T4. Nature 227: 680-655.
3. Candiano G, Bruschi M, Musante L, Santucci L, Ghiggeri GM, et al. (2004) Blue silver: a very sensitive colloidal Coomassie G-250 staining for proteome analysis. Electrophoresis 2004; 25:1327-1333.
4. Towbin H, Staehelin T, Gordon J (1979) Electrophoretic transfer of proteins from polyacrylamide gels to nitrocellulose sheets: procedure and some applications Proc Natl Acad Sci U S A 76: 4350-4354.
5. Mathias RA, Lim JW, Ji H, Simpson RJ (2009) in: Matthew J Peirce, RW editors Proteomics analysis of Membrane Proteins: Methods and Protocols. Peirce MJ, Wait R. Humana Press Totowa pp. 227- 242.
6. Wessel D, Flugge UI (1984) A method for the quantitative recovery of protein in dilute solution in the presence of detergents and lipids. Analytical Biochemistry 138: 141-143.
7. Lin Y, Zhou J, Bi, D., Chen P, Wang X et al. (2008) Sodium-deoxycholate-assisted tryptic digestion and identification of proteolytically resistant proteins Anal Biochem 377: 259–266.
8. Lin Y, Liu Y, Li J, Zhao Y, He Q et al. (2010) Evaluation and optimization of removal of an acid-insoluble surfactant for shotgun analysis of membrane proteome. Electrophoresis 31: 2705-2713.
